# Validation of a Bayesian Ebola Diagnostic Model Using Data from the Tenth Epidemic of 2018 to 2020 in the Democratic Republic of Congo

**DOI:** 10.3390/diagnostics16172828

**Published:** 2026-09-02

**Authors:** John Kamwina Kebela, Jean Nyandwe Kyloka, Junior Bulabula-Penge, Prince Kimpanga Diangs, Jack Kokolomami Hyombo, Godfroid Musema Mulakilwa, Dieudonné Mwamba Kazadi, Steve Ahuka-Mundeke, Rostin Mabela Makengo, Simon Ntumba Badibanga, Antoine Nkuba Ndaye, Olivier Mangapi Kinze, Berthe Barhayiga Nsimire, Etienne Bwira Mwokozi, Sylvain Munyanga Mukongo

**Affiliations:** 1 Department of Epidemiology and Biostatistics, School of Public Health, Faculty of Medicine, University of Kinshasa, Kinshasa P.O. Box 11850 Kinshasa I, Democratic Republic of the Congo; 2 Faculty of Medicine, Université Protestante au Congo (UPC), Kinshasa P.O. Box 4745, Democratic Republic of the Congo; bulabula.penge@upc.ac.cd; 3 National Institute for Biomedical Research (INRB), Kinshasa P.O. Box 1197, Democratic Republic of the Congo; 4 Department of Mathematics and Computer Science, Faculty of Science, University of Kinshasa, Kinshasa P.O. Box 190 Kinshasa XI, Democratic Republic of the Congo; 5 Educational and Vocational Guidance Department, Ilebo Higher Pedagogical Institute, Ilebo P.O. Box 198, Democratic Republic of the Congo; 6 Department of Anesthesia and Intensive Care, Cliniques Universitaires de Kinshasa (CUK), University of Kinshasa, Kinshasa P.O. Box 123 Kinshasa XI, Democratic Republic of the Congo; 7 Department of Management, School of Public Health, University of Kinshasa, Kinshasa P.O. Box 11850 Kinshasa I, Democratic Republic of the Congo

**Keywords:** subjective Bayesian model, validation, Democratic Republic of the Congo

## Abstract

**Background/Objectives**: Diagnosing Ebola virus disease (EVD) based solely on clinical signs remains challenging in settings where its manifestations overlap with other endemic infections. This study aimed to validate a Bayesian clinical diagnostic model to support decision-making for suspected EVD cases in resource-limited settings without access to laboratory confirmation. **Methods**: We conducted a retrospective analysis using data from the 10th Ebola outbreak in the Democratic Republic of the Congo (2018–2020). Clinical information from 450 suspected and laboratory-confirmed EVD cases was evaluated by seven epidemiological surveillance experts. Data were analyzed using descriptive statistics, binary logistic regression, discriminant analysis, and receiver operating characteristic (ROC) curves to assess diagnostic performance. **Results**: At the predefined 0.80 threshold, the SBM showed a sensitivity of 82.4%, specificity of 33.7%, PPV of 80.5%, and NPV of 36.5%. Balanced accuracy was 58.0%, with LR+ of 1.24 and LR− of approximately 0.52. Thus, although the model identified a relatively high proportion of RT-PCR confirmed cases, its ability to discriminate non-EVD cases was limited. At the symptom level, vomiting (*p* = 0.003), anorexia (*p* = 0.041), abdominal pain (*p* = 0.024), and muscle pain (*p* = 0.006) were independently linked to confirmed cases. **Conclusions**: This Bayesian model shows potential as a decision support tool for early identification of EVD in settings lacking laboratory capacity. Its use could improve case detection and support outbreak response in resource-limited contexts. The expert-derived SBM showed relatively high sensitivity but limited specificity in this selected outbreak cohort. These findings support further recalibration and independent validation rather than use of the model as a stand-alone diagnostic tool.

## 1. Introduction

Ebola virus disease (EVD) was first identified in 1976 during two simultaneous outbreaks: one in Nzara, in what was then southern Sudan (now South Sudan), and the other in Yambuku, in the Democratic Republic of the Congo (then Zaire). Since then, healthcare providers have faced challenges in clinically assessing patients with suspected EVD, as definitive confirmation requires laboratory testing rather than clinical information alone [1]. To date, 36 EVD epidemics have been recorded, mainly in Central and West Africa [2].

The Democratic Republic of the Congo (DRC) has experienced recurrent Ebola virus disease (EVD) outbreaks since 1976. The tenth EVD outbreak, which occurred from 2018 to 2020, was the longest and largest EVD outbreak recorded in the DRC, lasting 695 days and resulting in 3470 cases and 2280 deaths, corresponding to a case fatality rate of approximately 66% [3,4]. Individual-level analyses from the 2018–2020 EVD outbreak in the DRC have provided additional insight into the epidemiological characteristics and transmission dynamics of this major outbreak [5]. 

At the start of each epidemic and throughout the response period, healthcare providers are constantly at risk. For example, during the 1995 Ebola epidemic in the DRC, 80 of 315 (25%) healthcare workers contracted EVD [6].

Accurate clinical diagnosis of Ebola virus disease (EVD) is often difficult in outbreak-affected regions, where symptoms frequently overlap with other endemic conditions such as malaria, typhoid fever, Lassa fever, or other viral hemorrhagic fevers.

This problem has been highlighted by various authors [7]. Recent studies conducted during the tenth EVD outbreak in the DRC have demonstrated that clinical and epidemiological variables can be combined into prediction scores to improve the identification of EVD among suspected cases at triage [8]. External validation of an Ebola risk score in the DRC has also shown that predictive performance may decrease when a model is applied to a population different from the one in which it was originally developed, emphasizing the importance of independent validation [9]. The clinical criteria commonly used to identify a suspected case of Ebola include fever, unexplained bleeding, sudden death, and previous contact with a person with a suspected, probable, or confirmed case of Ebola [10]. However, A. Desclaux et al. [11] noted that the symptoms of EVD are not always present, and up to 10% of cases of EVD may not present with fever [12].

Previous studies have identified various clinical signs and symptoms associated with EVD in the DRC and West Africa based on different analytical approaches [13,14,15,16,17]. During the 2018 EVD outbreak in the Equateur province of the DRC, which began on May 8 and was declared over on July 24, epidemiologists reported that among confirmed or probable cases, the most frequently reported clinical signs were fever (40/42, 95%), intense general fatigue (37/41, 90%), and loss of appetite (37/41, 90%). Gastrointestinal symptoms were frequently reported, and 14/43 (33%) of patients reported hemorrhagic signs [18].

In the Ebola management center of Kailahun District, an isolated rural district in Sierra Leone, among 489 confirmed cases (51% male, median age 28 years), twenty-eight (6%) were healthcare workers: two physicians, 11 nurses, two laboratory technicians, seven community health workers, and six individuals from other professional categories. Over 50% of patients presented with fever, headache, abdominal pain, and diarrhea/vomiting [19].

In the Équateur Province outbreak investigation, the median age of confirmed or probable cases was 40 years (range 8–80), and 60% were male; the most common signs and symptoms included fever, severe fatigue, loss of consciousness, and loss of appetite [20].

A quantitative observational study of EVD was conducted in 2012 in Isiro, Haut-Uélé Province. Among 52 patients, the majority of patients at the Ebola treatment center (ETC) had a fever (55.6%) during their stay at the Ebola treatment unit [18]. In addition to fever, the main symptoms observed at the ETC included asthenia (82.4%), anorexia (82.4%), myalgia (70.6%), sore throat/difficulty swallowing (70.6%), arthralgia (76.5%), and nausea (70.6%). Gastrointestinal signs and symptoms (nausea, diarrhea, vomiting) (76.4%) and general aches and pains (94.1%) are common in patients admitted to the ETC [21].

As Mary-Anne Hartley emphasized, nonspecific symptoms of Ebola virus disease (EVD) pose a major challenge to triage and isolation efforts in Ebola treatment centers (ETCs). A better understanding of the statistical relevance of individual triage symptoms is essential in low-resource settings where rapid, laboratory-confirmed diagnoses are often unavailable [7]. Despite the potential value of clinical screening tools, laboratory confirmation remains essential for EVD diagnosis, with RT-PCR-based assays such as GeneXpert serving as the reference diagnostic approach during recent outbreaks in the DRC [22].

Uncertainty is a constant challenge in medical practice, where decisions must often be made with limited information [23]. In this context, a Bayesian model to support clinical assessment of Ebola has been developed and internally validated in the DRC [24]. This study aims to validate this subjective model of Ebola diagnosis using real data from the tenth epidemic in the DRC. The results will be compared with those from Mary Anne Hartley’s study on malaria-sensitive triage for Ebola [7].

## 2. Materials and Methods

### 2.1. Study Design and Data Collection

A retrospective cross-sectional diagnostic prediction study was conducted using secondary data from the 10th Ebola outbreak in the DRC (2018–2020). In addition, expert opinions were collected from epidemiological surveillance experts with experience in Ebola outbreak response in West Africa and during the 10th Ebola outbreak in the Democratic Republic of the Congo [24]. The Bayesian model was developed according to the subjective Bayesian approach proposed by De Dombal. The prior odds represented the experts’ initial estimate of the probability that a patient fulfilling the national suspected Ebola case definition actually had EVD before considering the predictive symptom groups. The prior odds were obtained through structured expert consensus using the nominal group technique rather than from the validation dataset. The initial prior odds value was 2.18 [24]. 

### 2.2. Bayesian Posterior Probability Calculation

Once the prior odds (QAPRI) and the likelihood ratio (LR) had been determined for each patient, the posterior odds (QAPO) were calculated according to Bayes’ theorem using the following relationship:

Posterior Odds (QAPO) = Likelihood Ratio (LR) × Prior Odds (QAPRI)

The posterior odds were subsequently converted into the posterior probability, which represents the updated probability of Ebola virus disease (EVD) after considering the patient’s clinical findings, using the following equation:

$$Posterior\;Probability=\frac{QAPO}{1+QAPO}$$

Patients with a posterior probability ≥0.80 were classified as SBM-positive, according to the threshold predefined during development of the original model.

During the original development of the subjective Bayesian model, candidate discrimination thresholds were evaluated at increments of 0.10 (0.1, 0.2, …, 0.8, and 0.9). The 0.8 category provided the best combination of sensitivity, specificity, PPV, and NPV and was therefore retained as the prespecified discrimination range. Because the original threshold grid was defined to one decimal place, the operational implementation used a more precise posterior probability value of 0.88, which lies within the 0.80–<0.90 interval represented by the original 0.8 cut-off category. This operational threshold was defined independently of the ROC analysis performed in the present validation dataset.

The sample size was determined according to recommendations for the development of multivariable diagnostic prediction models. Assuming a minimum of 20 events per predictor parameter and seven candidate predictor groups included in the model, at least 140 confirmed Ebola virus disease cases were required. Given an expected Ebola prevalence of approximately 50% in the study database, a minimum total sample size of 280 participants was estimated [25]. The final study included 450 patients, exceeding the minimum recommended sample size and ensuring adequate statistical power for model development and validation [26].

The study followed the Transparent Reporting of a Multivariable Prediction Model for Individual Prognosis or Diagnosis (TRIPOD) guidelines. Sample size adequacy was assessed according to the framework proposed by Riley et al. for prediction model development, and the final sample of 450 patients exceeded the minimum recommended size for robust model estimation and validation [27].

### 2.3. The Variables

The clinical information (signs and symptoms) of Ebola virus disease (EVD) identified by experts and the corresponding variables available in the database of the 10th EVD outbreak in the DRC are presented in Table 1.

### 2.4. Inclusion Criteria

The analytical cohort comprised 450 consecutively registered suspected EVD patients extracted from the national surveillance database. The cohort was not obtained by simple random sampling and should not be considered representative of all persons notified during the tenth EVD outbreak. Laboratory confirmation status and the variables required for the primary SBM evaluation were available for the included patients; however, missing observations remained for some secondary demographic, hospitalization, occupational, and individual symptom variables. Accordingly, denominators are reported separately where applicable. Of the 450 patients included in the analytical cohort, 346 (76.9%) were RT-PCR-positive. This proportion reflects the enriched composition of the analytical dataset rather than the positivity rate among all notified suspected cases during the outbreak. Consequently, prevalence-dependent performance measures, particularly PPV and NPV, should be interpreted in the context of this selected population. 

### 2.5. Data Entry and Analysis

The National Institute of Public Health (INSP) of the DRC provided the data in comma-separated value (CSV) format, which we exported to Microsoft Excel 2024 (Microsoft Corporation, Redmond, WA, USA) and IBM SPSS Statistics, version 32.0 (IBM Corp., Armonk, NY, USA), for data management and statistical analysis. Then, we conducted analyses, including binary logistic regression and discriminant analysis, to validate the Bayesian subjective model for the clinical diagnosis of Ebola. The reporting of the prediction model evaluation was guided, where applicable, by contemporary recommendations for transparent reporting of clinical prediction model studies [28].

### 2.6. Specimen Collection, Processing, and Laboratory Confirmation of Ebola Virus Disease

All suspected EVD cases were confirmed by real-time reverse transcription PCR (RT-PCR) at INRB field laboratories, following standard procedures established during the tenth EVD outbreak in the DRC. Venous blood samples in EDTA tubes were used for diagnosing living suspected patients, while oropharyngeal swabs were collected from deceased individuals during safe and dignified burials. After collection, specimens were transported under temperature-controlled conditions (2–8 °C) to the nearest INRB lab. Upon arrival, they were decontaminated, registered, and processed inside glovebox facilities by trained staff. All samples were tested with the GeneXpert Ebola RT-PCR assay (Cepheid), the reference method, in which a cycle threshold (Ct) value below 40 for the nucleoprotein (NP) target indicated a positive result, and Ct ≥ 40 or no detection indicated a negative result. Invalid results were retested with the same sample, and if repeat testing failed, a new specimen was collected. Following World Health Organization (WHO) and outbreak protocols, repeat RT-PCR tests were performed 48–72 h after symptom onset for patients with initial negative results within the first 72 h of illness, to reduce false negatives during early infection. 

### 2.7. Validation of the Subjective Bayesian Model (SBM) Against Real-World Cases

The subjective Bayesian model (SBM) for the clinical diagnosis of Ebola was validated against real-world cases from the same outbreak by comparing the subjective data from experts in Ebola diagnosis [24] with real data from the Ebola epidemic from 2018 to 2020. This comparison was performed to check the robustness and applicability of the model in a real epidemic context. The different parameters used to compare the Bayesian model with the actual Ebola cases were calculated using the following formulas:

$$S_{e}=\frac{a}{a+c}\times 100;\;S_{p}=\frac{d}{b+d}\times 100;\;PPV=\frac{a}{a+b}\times 100;\;NPV=\frac{d}{c+d}\times 100;\;OEV=\frac{a+d}{a+b+c+d}\times 100$$

a: True Positive (TP)/positive prediction AND disease truly present.

b: False positive (FP)/positive prediction BUT disease absent.

c: False negative (FN)/negative prediction BUT disease present.

d: True negative (TN)/negative prediction AND disease absent.

Se: sensitivity; Sp: specificity; PPV: positive predictive value; NPV: Negative Predictive Value; OEV: overall efficiency value.

### 2.8. Data Availability

The datasets analyzed during the current study are not publicly available due to national data-sharing regulations in the Democratic Republic of the Congo. However, they may be made available from the corresponding author upon reasonable request and with permission from the Ministry of Public Health.

Two distinct data sources were used in this study:

- Ebola outbreak surveillance data: Data from the 10th Ebola epidemic (2018–2020) were provided by the Direction Générale de la Lutte contre les Maladies (DGLM), Democratic Republic of the Congo (reference no. MS. DGLM/CM/046/MKD/2020). Requests for access to these data should be directed to Dr. Dieudonné Mwamba Kazadi (email: dieudonnemwambakazadi@gmail.com).
- Expert-generated dataset: The second dataset was generated by a panel of Ebola epidemiological surveillance experts during the development of the Bayesian clinical diagnostic model. Requests for access should be directed to Mr. John Kamwina Kebela (email: john.kamwina@unikin.ac.cd).

### 2.9. Study Limitations

The study evaluated the model using surveillance data from the same outbreak. This validation does not establish transportability or generalizability to other Ebola outbreaks or populations. Future studies should perform independent external validation using datasets collected from different outbreaks, geographical settings, or time periods before the model can be recommended for routine clinical implementation.

## 3. Results

### 3.1. Sociodemographic Characteristics of the Ebola-Positive Patients

In Table 2, among the 450 patients included in the study, 346 (76.9%) tested positive for EVD. Among confirmed cases with available hospitalization data (n = 338), 246 (72.8%) were hospitalized. Denominators vary across variables due to missing data in the surveillance forms. The mean age was 25.75 years, with a margin of error of ±1.74 years (95% CI: 24.01–27.49). The median age was 24.50 years (95% CI: 22.50–26.00).

In the bivariate analysis of sociodemographic data, several variables were found to be statistically significantly associated with laboratory-confirmed Ebola virus infection. Patient age was significantly associated with confirmation status (*p* = 0.011). Housewives (OR = 2.27, 95% CI: 1.04–4.93; *p* = 0.036) and participants classified under other occupations (OR = 2.51, 95% CI: 1.10–5.70; *p* = 0.022) had higher odds of laboratory-confirmed EVD. Conversely, children had lower odds of laboratory-confirmed EVD (OR = 0.53, 95% CI: 0.33–0.84; *p* = 0.008). No statistically significant association was observed for the remaining occupational categories. These findings suggest that, within our sample, age and membership in these occupational groups are associated with a higher likelihood of a positive Ebola virus laboratory test (Table 2).

### 3.2. Validation of the Subjective Bayesian Model for Ebola Diagnosis Against Real-World Cases from the Same Outbreak 

In Table 3, five factors were significantly associated with Ebola virus disease (EVD) positivity. Hemorrhagic factors (F1) were strongly associated with a reduced likelihood of EVD positivity (*p* < 0.001; OR = 0.218; 95% CI: 0.132–0.358). In contrast, neurological factors (F2) (*p* = 0.020; OR = 1.695; 95% CI: 1.083–2.653), digestive factors (F3) (*p* = 0.010; OR = 1.932; 95% CI: 1.163–3.210), pain-related factors (F4) (*p* < 0.001; OR = 2.148; 95% CI: 1.347–3.426), and general factors (F5) (*p* < 0.001; OR = 3.249; 95% CI: 1.733–6.090) were all significantly associated with an increased probability of EVD positivity.

Table 4 shows the two-by-two contingency table comparing the subjective Bayesian model (SBM) with actual EVD cases. At the predefined 0.80 threshold, the SBM yielded a sensitivity of 82.4%, specificity of 33.7%, PPV of 80.5%, and NPV of 36.5%. Balanced accuracy was 58.0%, the Youden index was 0.16, LR+ was 1.24, and LR− was approximately 0.52. Although sensitivity was relatively high, the low specificity, NPV, and LR+ indicate limited diagnostic discrimination at this threshold. Overall accuracy was 71.1%; however, because 76.9% of the analytical cohort was RT-PCR-positive, overall accuracy alone provides a potentially misleading assessment of model performance. 

In Table 5, the subjective Bayesian model is compared with the logistic regression model (LRM) using the same cut-off value of 0.8 across 450 cases. Overall, the SBM applied to real cases appears to outperform the SBM-LRM combination for EVD detection.

Agreement between the SBM and the LRM was assessed using Cohen’s kappa coefficient. The observed agreement was 63.6%, whereas the agreement expected by chance was 56.8%, resulting in a Cohen’s kappa coefficient of 0.16, indicating slight agreement beyond chance. Therefore, the comparison between the SBM and the LRM should be interpreted as an assessment of concordance between two predictive models rather than an evaluation of diagnostic accuracy.

Table 6 compares the logistic regression model with real EVD cases. The SBM applied to real cases showed higher sensitivity (82.4%) than the LRM (71.7%), indicating better detection of confirmed cases. However, specificity was considerably lower (33.7% vs. 71.2%), reflecting a higher rate of false positives. The PPV was also lower in the SBM (80.5%) compared with the LRM (89.2%), suggesting reduced reliability of positive predictions. Similarly, the NPV was lower (36.5% vs. 43.0%), indicating weaker performance at identifying true negatives. Despite these differences, overall efficiency value accuracy was comparable between the two models (71.1% vs. 71.6%).

Table 7 presents the results of the logistic regression model, including all seven factors for predicting Ebola virus disease (EVD) diagnosis. In this model, only hemorrhagic factors (*p* < 0.001), pain-related factors (*p* = 0.010), general factors (*p* < 0.001), and epidemiological link factors (*p* = 0.040) were significantly associated with a confirmed clinical diagnosis of EVD.

**Figure 1:**
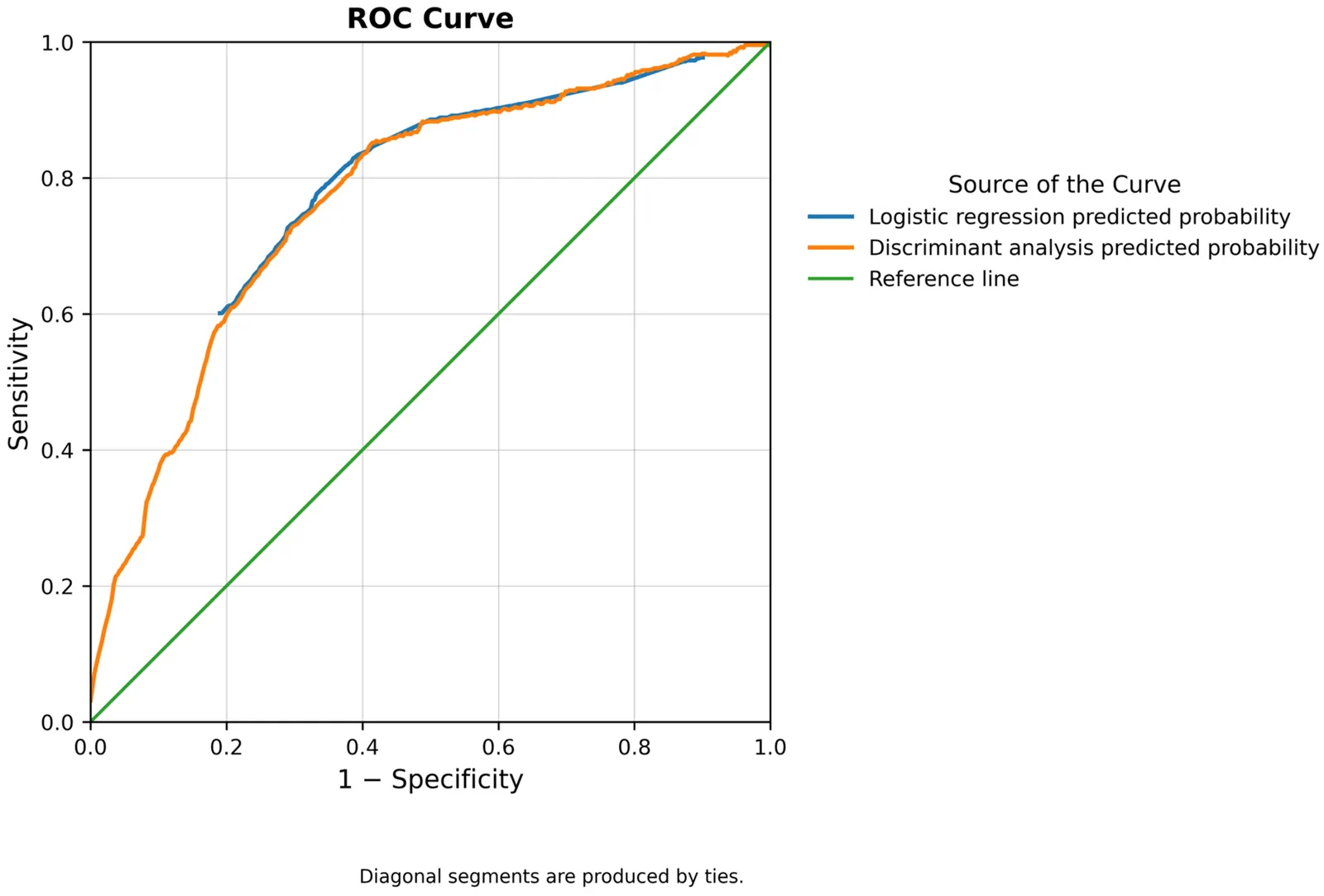
ROC curve of the predicted probability of Ebola clinical diagnosis via logistic regression and discriminant analysis.

Receiver operating characteristic (ROC) curves were constructed to evaluate the discriminatory performance of the logistic regression and discriminant analysis models. The area under the ROC curve (AUC) was 0.772 for the logistic regression model and 0.770 for the discriminant analysis model, indicating acceptable discrimination for both models. The corresponding ROC curves are presented in Figure 1. These findings reflect the predictive performance of the logistic regression and discriminant analysis models and should not be interpreted as a direct evaluation of the subjective Bayesian model.

The positive likelihood ratio (LR+) was 1.24, indicating that a positive SBM result increased the odds of Ebola virus disease only marginally. This value suggests limited diagnostic utility and is unlikely to produce a clinically meaningful change in the pretest probability of disease.

Likelihood ratios are independent of disease prevalence and provide an estimate of how much a test result changes the odds of disease. In contrast, predictive values depend on disease prevalence.

Using the prespecified operational SBM threshold of 0.88, derived within the original 0.8 < 0.9 discrimination range, the model yielded a sensitivity of 82.4% and a specificity of 33.7%. In a separate exploratory ROC analysis using the continuous posterior probabilities, the SBM achieved an AUC of 0.683 (95% CI: 0.633–0.733). The maximum Youden index was observed at a posterior probability of approximately 0.903, corresponding to a sensitivity of 37.6% and a specificity of 99.0%. This ROC-derived threshold was considered exploratory and was not used to redefine the prespecified SBM classification rule.

**Figure 2:**
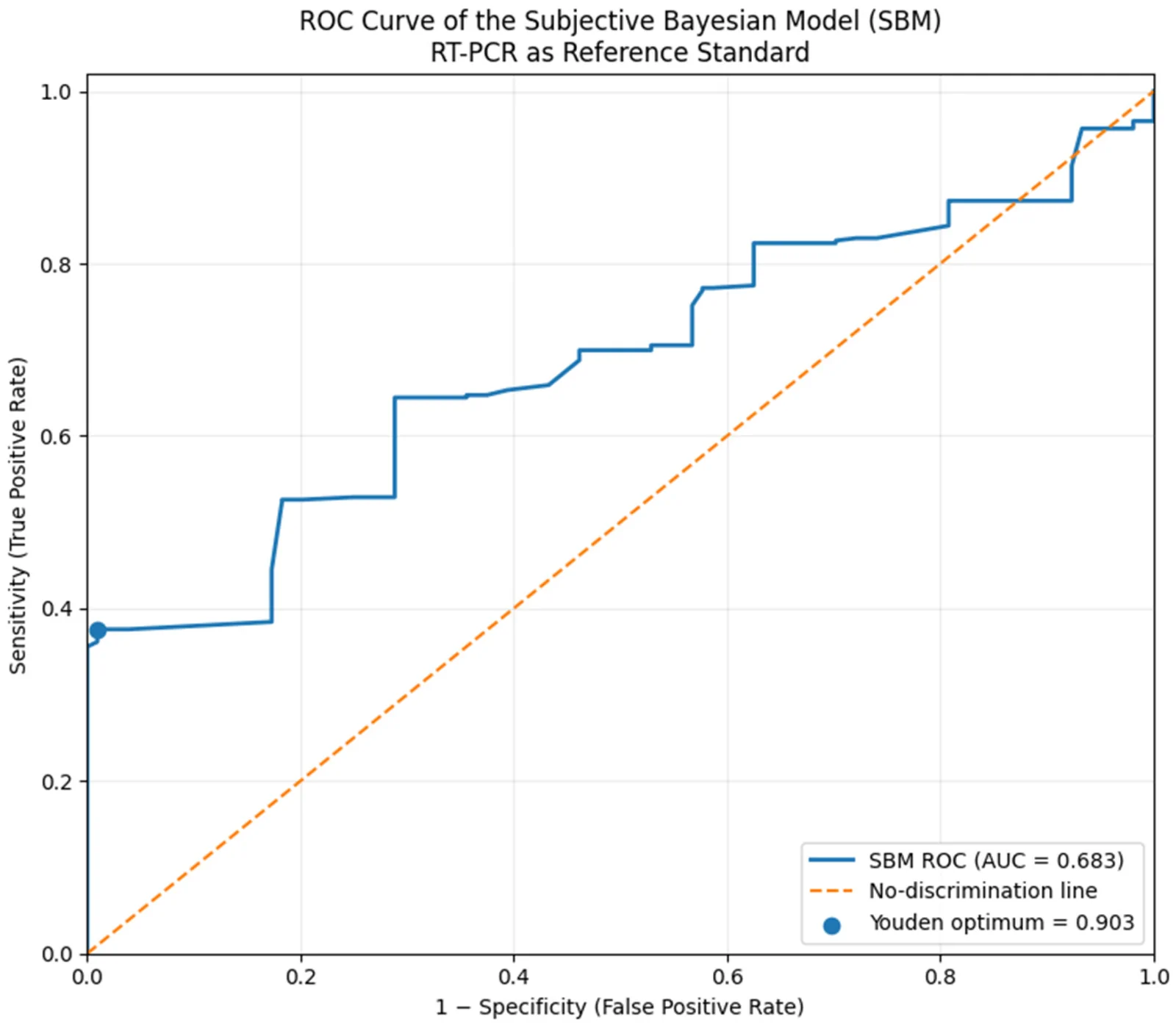
Receiver operating characteristic (ROC) curve of the subjective Bayesian model for predicting RT-PCR-confirmed Ebola virus disease.

The ROC curve was constructed from the continuous patient-level posterior probabilities generated by the SBM (Figure 2). The area under the curve was 0.683 (95% CI: 0.633–0.733). RT-PCR was used as the reference standard.

In the dataset analyzed, the most frequently reported symptoms among laboratory-confirmed EVD cases were fatigue (82.7%), anorexia (67.3%), fever (60.7%), vomiting (47.3%), and diarrhea (35.7%), consistent with the established clinical presentation of Ebola virus disease (Table 8).

Bivariate analysis identified several symptoms significantly associated with laboratory-confirmed EVD, including fatigue (*p* = 0.001), anorexia (*p* = 0.001), abdominal pain (*p* = 0.003), muscle pain (*p* < 0.001), joint pain (*p* = 0.037), and headache (*p* = 0.006) (Table 9).

In the multivariate analysis, vomiting (*p* = 0.003), anorexia (*p* = 0.041), abdominal pain (*p* = 0.024), and muscle pain (myalgia) (*p* = 0.006) remained independently and significantly associated with laboratory-confirmed EVD. These results indicate that, after adjustment for other model variables, the four symptoms are independently associated with an increased likelihood of Ebola virus infection in the study population (Table 10).

## 4. Discussion

The sociodemographic characteristics of laboratory-confirmed Ebola virus disease (EVD) cases in the present study were generally consistent with those reported during previous African outbreaks. Female patients represented a slight majority of confirmed cases, and the age distribution was comparable to that reported by Qin and Barry. Although some occupational categories, including housewife, child, and other profession, showed higher odds of laboratory-confirmed infection in the univariate analysis, these findings should be interpreted cautiously because they may reflect differences in exposure patterns rather than independent risk factors (Table 2).

The sociodemographic profile of Ebola-positive patients in our study was broadly consistent with previous reports of outbreaks [13]. For example, Qin and al. reported a female proportion of 54.1% and a median age of 28 years among confirmed cases, which are comparable to our findings; however, they did not report a higher female fatality rate [29]. Where sex differences are discussed, they should be interpreted in the context of gendered exposure patterns and access to care, as described in gender-focused risk analyses. In the present cohort, healthcare professionals (including nurses, laboratory technicians, and surface technicians) accounted for 3.4% of the Ebola-positive patients. Bah et al. reported that 38% of healthcare workers exposed to Ebola experienced nosocomial transmission, underscoring the occupational risk in outbreak settings [13]. However, our results differ from those of M. Barry with respect to age, sex, and occupation [15].

Several clinical manifestations, including fatigue, anorexia, abdominal pain, headache, myalgia, and arthralgia, were significantly associated with laboratory-confirmed EVD in the univariate analysis. These findings are in agreement with previous studies conducted in Sierra Leone, Guinea, Equateur Province, and Isiro, where gastrointestinal and constitutional symptoms predominated among confirmed patients.

After adjustment in the multivariable logistic regression model, only vomiting, anorexia, abdominal pain, and muscle pain remained independently associated with laboratory-confirmed EVD. This suggests that several symptoms observed in the univariate analysis were explained by confounding between clinical manifestations.

Multivariable logistic regression was performed to identify clinical variables independently associated with laboratory-confirmed EVD. Four symptom groups remained statistically significant after adjustment. However, these regression coefficients were not used to construct the Bayesian diagnostic model; instead, they served to explore independent clinical associations within the validation dataset.

The clinical symptoms and signs observed among the Ebola-positive patients herein are consistent with those reported in Barry et al. [18], Feldmann and Geisbert [20], Bwaka et al. [30], Moole et al. [14], and Lado et al. [15]. In particular, hemorrhagic signs, although significant (*p* < 0.001), were less frequent in this study than in previous studies, thus corroborating Bwaka’s findings that these signs are rare [30]. Marta Lado reported that the combination of symptoms such as fever, intense fatigue, vomiting, and diarrhea increased the probability of EVD [15]. We also found significant factors consistent with Jain’s observations, such as neurological (*p* = 0.020; OR = 1.695) and general (*p* < 0.001; OR = 3.249) factors, thus emphasizing the importance of advanced symptoms such as confusion and jaundice [16].

In a study carried out in Equateur Province in April–May 2018 (DR Congo) and another conducted in Isiro, Haut Uélé Province, almost the same symptoms were observed as in our study [18,21]. In the study conducted in Isiro, differential diagnoses of EVD included malaria (28.3%), intestinal parasitosis (10.9%), and infectious syndrome (10.9%) [21].

Similarly, in a study of 90 patients infected with EVD in Conakry, the most frequent clinical signs were asthenia (80%), fever (72%), nausea/vomiting (60%), diarrhea (34%), and myalgia (23%) [31]. A total of 14% of patients presented with respiratory distress, and 26% presented with hemorrhagic signs. Malaria (72%) and diabetes (2%) were the most common comorbidities [31].

Although fever was reported in almost all literature reviews and observed in our study as a symptom that can guide the diagnosis of EVD, 10% of EVD cases do not present with fever [32]. In a study of the annual incidence of malaria in Sierra Leone, malaria was more common among patients triaged for suspected EVD than among those with confirmed EVD [7].

Our findings show that symptoms such as fatigue, anorexia, abdominal pain, muscle pain, joint pain, and headache are significantly more frequent among patients with laboratory-confirmed Ebola virus disease. This suggests that these clinical manifestations may form part of the symptomatic profile associated with confirmed Ebola infection in our sample. However, as the analysis is bivariate and unadjusted, these associations cannot be interpreted as causal and may be influenced by confounding or other biases. Therefore, we performed multivariate analyses (odds ratios with confidence intervals) (Table 10).

The multivariate results indicate that vomiting, anorexia, abdominal pain, and muscle pain were independently associated with laboratory-confirmed Ebola virus infection in our sample. Association does not necessarily imply predictive contribution; formal assessment of predictive utility requires model performance measures (e.g., calibration and discrimination) and evaluation of clinical utility.

An important finding of the present evaluation was the discordance between the relative importance assigned to F1 and F6 in the original expert-derived model and their empirical associations in the outbreak dataset. Hemorrhagic manifestations, although considered a major positive diagnostic factor by experts, were substantially less frequent among RT-PCR-confirmed patients than among non-confirmed patients and were inversely associated with confirmation. Likewise, epidemiological linkage, which received substantial weight in the expert-derived model, did not discriminate confirmed from non-confirmed cases in the univariate analysis and showed an inverse association after multivariable adjustment. These discrepancies suggest that expert-elicited diagnostic weights may not be directly transportable to heterogeneous field surveillance populations. They also reinforce the need for recalibration and independent validation of the SBM before clinical implementation.

Validation against real-world cases from the same outbreak showed that the model could support triage decisions, with a sensitivity of 82.4% and a positive predictive value of 80.5% at a 0.8 cut-off. However, specificity was low, and these findings should be interpreted as performance within the same outbreak dataset rather than independent external validation.

Validation of the SBM against real-world cases from the same outbreak showed that the model performed well at detecting Ebola cases, with a sensitivity of 82.4%, a PPV of 80.5%, and an OEV of 71.1%. Recent evidence from a systematic review and meta-analysis of antigen-based rapid diagnostic tests further underscores the importance of carefully considering sensitivity, specificity, and the intended clinical context when evaluating tools for EVD diagnosis [33]. A comparison with field results shows that our model outperformed the LRM in terms of sensitivity. Logistic regression analysis confirmed the significance of four factors: hemorrhagic, painful, general, and epidemiological factors. Other predictive approaches, including machine learning models, have also been investigated in EVD and have highlighted the importance of model validation and updating when prediction tools are transferred across populations and outbreak settings [34]. 

The ROC analysis evaluated the discriminative ability of the multivariable logistic regression model and the discriminant analysis rather than the Bayesian model itself. Both approaches showed acceptable discrimination (AUC ≈ 0.77), indicating moderate ability to distinguish laboratory-confirmed EVD from non-EVD cases. These results should therefore be interpreted as supporting the statistical performance of the regression-based classifiers and not as direct evidence of the diagnostic accuracy of the Bayesian model.

Study limitations and methodological clarification: A key limitation of this study is the nature of the model development and validation process. The Bayesian diagnostic model was initially constructed using expert-generated subjective data rather than empirical patient data, introducing potential bias and limiting reproducibility. While this approach may be justified in emergency or low-resource settings where rapid decision-making is required and laboratory confirmation is unavailable, it is not a standard epidemiological method. Furthermore, the validation performed in this study does not constitute true external validation. External validation requires testing the model on an independent population or dataset from a different setting, time period, or demographic group. In this manuscript, validation was conducted using real-world data from the same epidemic, a form of internal or temporal validation. These limitations highlight the need for future evaluation on independent cohorts to assess generalizability and reduce bias.

Bayes’ theorem is comparable to epidemiological measurements, and there is a direct connection between diagnostic tests and Bayesian statistics, enabling the calculation of PPV and NPV [25].

## 5. Conclusions

In this study, several clinical manifestations, including vomiting, anorexia, abdominal pain, and muscle pain, were independently associated with laboratory-confirmed Ebola virus disease (EVD) in patients from the tenth EVD outbreak in the Democratic Republic of the Congo. These findings are consistent with the clinical spectrum reported in previous outbreaks and highlight the predominance of gastrointestinal and constitutional symptoms among confirmed EVD cases.

The subjective Bayesian model (SBM), developed from expert-derived probabilities, demonstrated high sensitivity for identifying laboratory-confirmed EVD cases within the study population, suggesting potential utility as a triage support tool in outbreak settings where rapid clinical decision-making is required and laboratory confirmation may be delayed. However, the model showed limited specificity and only modest discriminative performance, indicating that it should not be considered a stand-alone diagnostic tool.

Multivariable logistic regression identified several symptom groups independently associated with laboratory-confirmed EVD, while ROC analysis demonstrated acceptable discrimination for the regression-based classifiers (AUC ≈ 0.77). These findings support the statistical validity of the regression models but should not be interpreted as direct evidence of the diagnostic accuracy of the Bayesian model.

Importantly, the present validation was conducted using real-world data from the same outbreak and therefore represents internal rather than true external validation. Consequently, the generalizability of the model to other outbreaks, populations, or epidemiological contexts remains uncertain.

Future studies should focus on prospective and external validation using independent datasets from different Ebola outbreaks and geographical settings. Such investigations are necessary to assess calibration, discrimination, clinical utility, and transportability before the model can be considered for broader implementation in EVD surveillance and clinical triage systems. 

## Figures and Tables

**Table 1:** Comparison of expert-identified factors with variables available in the 2019–2020 Ebola virus disease database in the Democratic Republic of the Congo.

No.	Clinical/Epidemiological Factors in Ebola Patients (Expert Opinion)	Expert-Derived Clinical/Epidemiological Factor Groups	Variables Available in the North Kivu–South Kivu–Ituri EVD Database (5 July 2020)
1	Hemorrhagic Signs (F1)	1. Hematemesis. 2. Red eyes (conjunctival injection). 3. Gingival bleeding. 4. Epistaxis. 5. Genital bleeding. 6. Miscarriage/abortion. 7. Bleeding from injection sites. 8. Melena. 9. Hemoptysis.	1. Unexplained bleeding. 2. Gum bleeding. 3. Injection site bleeding. 4. Nose bleeding. 5. Blood in stool. 6. Hematemesis. 7. Bloody vomiting. 8. Hemoptysis. 9. Vaginal bleeding. 10. Skin bleeding. 11. Hematuria. 12. Other bleeding.
2	Neurological Signs (F2)	1. Coma. 2. Convulsions. 3. Headache.	1. Headache. 2. Eye pain. 3. Unconsciousness. 4. Confusion or disorientation.
3	Gastrointestinal Signs (F3)	1. Diarrhea. 2. Vomiting. 3. Anorexia (loss of appetite). 4. Abdominal pain. 5. Epigastric pain.	1. Vomiting. 2. Diarrhea. 3. Anorexia. 4. Abdominal pain. 5. Hiccups.
4	Pain Syndrome (F4)	1. Myalgia. 2. Arthralgia. 3. Chest pain.	1. Chest pain. 2. Muscle pain. 3. Joint pain.
5	General Signs (F5)	1. Asthenia (general weakness). 2. Fever. 3. Dehydration. 4. Jaundice (icterus).	1. Fever. 2. Body temperature. 3. Fatigue. 4. Difficulty swallowing (dysphagia). 5. Sore throat. 6. Jaundice. 7. Conjunctivitis. 8. Skin rash.
6	Epidemiological Link (F6)	1. Contact with a confirmed case, probable case, or sick/dead animal. 2. Participation in an unsafe burial of a probable or confirmed case. 3. Unprotected sexual intercourse with an Ebola survivor.	1. Contact with a case. 2. Funeral attendance. 3. Contact with sick or dead animals.
7	Respiratory Signs (F7)	1. Dyspnea (shortness of breath). 2. Cough.	1. Cough. 2. Difficulty breathing.

NOTA: The grouping shown here distinguishes the original expert-derived factor definitions from variables available in the surveillance database. Availability of an additional symptom in the surveillance database does not imply that it was part of the original Bayesian model.

**Table 2:** Bivariate analysis of sociodemographic characteristics and Ebola laboratory results.

Sociodemographic Characteristic	Confirmed n (%)	Not Confirmed n (%)	Total n (%)	OR	95% CI	*p*-Value
**Patient sex**						
Male	166 (48.0)	53 (51.5)	219 (48.8)	0.87	[0.560; 1.351]	0.54
Female	180 (52.0)	50 (48.5)	230 (51.2)	1	-	
Total	346 (100.0)	103 (100.0)	449 (100.0)			
**Age of patient**						
0–5	63 (18.2)	31 (29.8)	94 (20.9)	1.91	[1.156; 3.149]	**0.011**
6 and older	283 (81.8)	73 (70.2)	356 (79.1)	1		
Total	346 (100.0)	104 (100.0)	450 (100.0)			
**Patient’s profession/Status**						
Farmer	86 (24.9)	31 (29.8)	117 (26.0)	0.78	[0.48; 1.27]	0.311
Butcher	6 (1.7)	1 (1.0)	7 (1.6)	1.82	[0.22; 15.27]	1.000
Miner	2 (0.6)	1 (1.0)	3 (0.7)	0.60	[0.05; 6.67]	0.546
Religious leader	5 (1.4)	2 (1.9)	7 (1.6)	0.75	[0.14; 3.91]	0.665
Housewife	55 (15.9)	8 (7.7)	63 (14.0)	2.27	[1.04; 4.93]	**0.036**
Pupil/student	21 (6.1)	6 (5.8)	27 (6.0)	1.06	[0.41; 2.69]	1.000
Child	83 (24.0)	39 (37.5)	122 (27.1)	0.53	[0.33; 0.84]	**0.008**
Traditional healer	2 (0.6)	2 (1.9)	4 (0.9)	0.30	[0.04; 2.13]	0.230
Company	11 (3.2)	2 (1.9)	13 (2.9)	1.68	[0.37; 7.68]	0.741
Carrier	11 (3.2)	3 (2.9)	14 (3.1)	1.11	[0.30; 4.04]	1.000
Health worker	11 (3.2)	2 (1.9)	13 (2.9)	1.68	[0.37; 7.68]	0.741
Other profession	53 (15.3)	7 (6.7)	60 (13.3)	2.51	[1.10; 5.70]	**0.022**
Total	346 (100.0)	104 (100.0)	450 (100.0)			
**The case is a contact**						
Yes	240 (69.4)	72 (69.2)	312 (69.3)	1.006	[0.626; 1.618]	0.979
No	106 (30.6)	32 (30.8)	138 (30.7)	1	-	
Total	346 (100.0)	104 (100.0)	450 (100.0)			

Bold indicates statistically significant *p*-values (*p* < 0.05).

**Table 3:** Factors associated with EVD based on Ebola laboratory results.

Factors Associated with EVD	Confirmed EVD n (%)	Not Confirmed EVD n (%)	Total n (%)	OR	95% CI	*p*-Value
**F1: Bleeding factors**						
**Yes**	46 (13.3)	43 (41.3)	89 (19.8)	0.218	[0.132; 0.358]	<0.001
**No**	300 (86.7)	61 (58.7)	361 (80.2)	1	-	
**Total**	346 (100.0)	104 (100.0)	450 (100.0)			
**F2: Neurological factors**						
**Yes**	178 (51.4)	40 (38.5)	218 (48.4)	1.695	[1.083; 2.653]	0.020
**No**	168 (48.6)	64 (61.5)	232 (51.6)	1	-	
**Total**	346 (100.0)	104 (100.0)	450 (100.0)			
**F3: Digestive factors**						
**Yes**	286 (82.7)	74 (71.2)	360 (80.0)	1.932	[1.163; 3.210]	0.010
**No**	60 (17.3)	30 (28.8)	90 (20.0)	1	-	
**Total**	346 (100.0)	104 (100.0)	450 (100.0)			
**F4: Pain factors**						
**Yes**	169 (48.8)	32 (30.8)	201 (44.7)	2.148	[1.347; 3.426]	0.001
**No**	177 (51.2)	72 (69.2)	249 (55.3)	1	-	
**Total**	346 (100.0)	104 (100.0)	450 (100.0)			
**F5: General factors**						
**Yes**	321 (92.8)	83 (79.8)	404 (89.8)	3.249	[1.733; 6.090]	<0.001
**No**	25 (7.2)	21 (20.2)	46 (10.2)	1	-	
**Total**	346 (100.0)	104 (100.0)	450 (100.0)			
**F6: Epidemiological link factors**						
**Yes**	248 (71.7)	74 (71.2)	322 (71.6)	1.026	[0.632; 1.665]	0.918
**No**	98 (28.3)	30 (28.8)	128 (28.4)	1	-	
**Total**	346 (100.0)	104 (100.0)	450 (100.0)			
**F7: Respiratory factors**						
**Yes**	102 (29.5)	30 (28.8)	132 (29.3)	1.031	[0.636; 1.672]	0.901
**No**	244 (70.5)	74 (71.2)	318 (70.7)	1	-	
**Total**	346 (100.0)	104 (100.0)	450 (100.0)			

**Table 4:** Comparison of SBM with real cases of EVD.

	Diagnosis of EVD	Real Cases of EVD		Total
		EVD+	EVD−	
**SBM (0** **.** **8)**	EVD+	285	69	354
	EVD−	61	35	96
	Total	346	104	450

**Table 5:** Agreement between the SBM and the LRM assessed using Cohen’s kappa coefficient.

	Diagnosis of EVD	LRM/COP (0.8)		Total
		EVD+	EVD−	
**SBM (0** **.** **8)**	EVD+	234	120	354
	EVD−	44	52	96
	Total	278	172	450

**Table 6:** Comparison of the LRM with real cases of EVD.

	Diagnosis of EVD	Real Cases of EVD		Total
		EVD+	EVD−	
**LRM. (0** **.** **8)**	EVD+	248	30	278
	EVD−	98	74	172
	Total	346	104	450

**Table 7:** Estimation of the diagnostic prediction model via logistic regression.

Variables in the Equation	B	E.S	Wald	Sig.	Exp(B)	95% Confidence Interval for EXP(B)	
						Lower	Upper
Bleeding factors	−2.07	0.29	49.47	<0.001	0.13	0.07	0.23
Neurological factors	0.50	0.27	3.55	0.06	1.65	0.98	2.78
Digestive factors	0.42	0.34	1.56	0.21	1.53	0.79	2.97
Pain factors	0.72	0.28	6.58	0.01	2.06	1.19	3.58
General factors	1.37	0.41	11.18	<0.001	3.92	1.76	8.73
Epidemiological link factors	−0.62	0.29	4.45	0.04	0.54	0.30	0.96
Respiratory factors	−0.21	0.29	0.54	0.46	0.81	0.46	1.42
Constant	0.23	0.34	0.46	0.50	1.26		

**Table 8:** Estimation of the diagnostic prediction model via logistic regression.

Symptoms and Signs	Not Confirmed EVD (%)	Confirmed EVD (%)
Fatigue	76.1	82.7
Anorexia (Loss of Appetite)	55.7	67.3
Fever	68.2	60.7
Headache	39.8	50.3
Vomiting	60.2	47.3
Abdominal Pain	30.7	43.2
Joint Pain	28.4	36.0
Diarrhea	40.9	35.7
Muscle Pain	14.8	31.3
Other Symptoms	22.7	23.2
Cough	23.9	20.5
Chest Pain	15.9	19.3
Difficulty Swallowing	19.3	17.9
Sore Throat	20.5	16.7
Unexplained Bleeding	48.9	13.7
Difficulty Breathing	14.8	13.7
Conjunctivitis	6.8	7.1
Bleeding Stools	21.6	5.4
Hiccups	8.0	5.1
Jaundice	2.3	4.2
Bleeding Gums	11.4	3.9
Injector Bleeding	1.1	2.7
Rash	5.7	2.7
Unconsciousness	10.2	2.7
Hematemesis	26.1	2.4
Vomiting Blood	2.3	2.4
Confused or Disoriented at Any Time	3.4	2.4
Nosebleed	12.5	2.1
Eye Pain	0.0	2.1
Vaginal Bleeding	0.0	0.9
Bleeding Urine	0.0	0.9
Other Bleeding	1.1	0.9
Coughing Blood	6.8	0.6

Note: Percentages were calculated based on the total number of patients (N = 450). Multiple responses were allowed; therefore, the sum of percentages may exceed 100%.

**Table 9:** Clinical symptoms and laboratory findings in individuals tested for Ebola virus disease (n = 450).

Symptoms/ Signs	Not Confirmed EVD		Confirmed EVD		Total		OR	95% CI	*p*-Value
	N	%	n	%	n	%			
**Fever**									
No	44	42.3	142	41.0	186	41.3	1		0.818
Yes	60	57.7	204	59.0	264	58.7	1.054	[0.676–1.643]	
Total	104	100.0	346	100.0	450	100.0			
**Vomiting**									
No	51	49.0	187	54.0	238	52.9	1		0.370
Yes	53	51.0	159	46.0	212	47.1	0.818	[0.528–1.269]	
Total	104	100.0	346	100.0	450	100.0			
**Diarrhea**									
No	68	65.4	226	65.3	294	65.3	1		0.990
Yes	36	34.6	120	34.7	156	34.7	1.003	[0.633–1.590]	
Total	104	100.0	346	100.0	450	100.0			
**Fatigue**									
No	37	35.6	68	19.7	105	23.3	1		**0.001**
Yes	67	64.4	278	80.3	345	76.7	2.258	[1.395–3.653]	
Total	104	100.0	346	100.0	450	100.0			
**Loss of appetite/Anorexia**									
No	55	52.9	120	34.7	175	38.9	1		**0.001**
Yes	49	47.1	226	65.3	275	61.1	2.114	[1.356–3.296]	
Total	104	100.0	346	100.0	450	100.0			
**Abdominal pain**									
No	77	74.0	201	58.1	278	61.8	1		**0.003**
Yes	27	26.0	145	41.9	172	38.2	2.057	[1.263–3.350]	
Total	104	100.0	346	100.0	450	100.0			
**Muscle pain/Myalgia**									
No	91	87.5	241	69.7	332	73.8	1		**0.000**
Yes	13	12.5	105	30.3	118	26.2	3.050	[1.633–5.696]	
Total	104	100.0	346	100.0	450	100.0			
**Joint pain/Arthralgia**									
No	79	76.0	225	65.0	304	67.6	1		**0.037**
Yes	25	24.0	121	35.0	146	32.4	1.699	[1.030–2.805]	
Total	104	100.0	346	100.0	450	100.0			
**Headache**									
No	69	66.3	177	51.2	246	54.7	1		**0.006**
Yes	35	33.7	169	48.8	204	45.3	1.882	[1.191–2.976]	
Total	104	100.0	346	100.0	450	100.0			

**Table 10:** Factors associated with positive Ebola virus disease by logistic regression.

Variables of the Equation	B	E.S	Wald	ddl	*p*-Value	OR	95% Confidence Interval	
							Lower	Upper
Fever	−0.008	0.244	0.001	1	0.974	0.992	0.615	1.6
Vomiting	0.855	0.289	8.772	1	0.003	2.352	1.335	4.142
Diarrhea	0.097	0.288	0.114	1	0.735	1.102	0.627	1.937
Fatigue	−0.482	0.305	2.492	1	0.114	0.618	0.34	1.123
Anorexia (loss of appetite)	−0.553	0.271	4.17	1	0.041	0.575	0.338	0.978
Abdominal pain	−0.645	0.285	5.114	1	0.024	0.525	0.3	0.918
Muscle pain	−1.043	0.383	7.416	1	0.006	0.352	0.166	0.746
Joint pain	0.278	0.321	0.75	1	0.387	1.32	0.704	2.476
Headache	−0.478	0.255	3.511	1	0.061	0.62	0.376	1.022
Constant	2.417	0.376	41.222	1	0	11.211		

## Data Availability

The data used in this study were obtained from two distinct sources. Ebola outbreak surveillance data from the 10th Ebola epidemic (2018–2020) in the Democratic Republic of the Congo were provided by the Direction Générale de la Lutte contre les Maladies (DGLM) under reference number MS. DGLM/CM/046/MKD/2020. These data are not publicly available due to ethical and confidentiality restrictions but may be obtained upon reasonable request and with permission from the DGLM. Requests should be directed to Dr. Dieudonné Mwamba Kazadi (email: dieudonnemwambakazi@gmail.com). The second dataset was generated by a panel of experts in Ebola epidemiological surveillance during the development of the Bayesian clinical diagnostic model. These data are also not publicly available but may be accessed upon reasonable request from the corresponding author or directly from Dr. John Kamwina Kebela (email: john.kamwina@unikin.ac.cd).

## Abbreviations

The following abbreviations are used in this manuscript:

AUC	Area Under the Curve
CI	Confidence Interval
Ct	Cycle Threshold
DGLM	Direction Générale de la Lutte contre les Maladies
DRC	Democratic Republic of the Congo
EDTA	Ethylenediaminetetraacetic Acid
ETC	Ebola Treatment Center
EVD	Ebola Virus Disease
FN	False Negative
FP	False Positive
INRB	Institut National de Recherche Biomédicale
INSP	National Institute of Public Health
LRM	Logistic Regression Model
NP	Nucleoprotein
NPV	Negative Predictive Value
OEV	Overall Efficiency Value
OR	Odds Ratio
PPV	Positive Predictive Value
ROC	Receiver Operating Characteristic
RT-PCR	Real-Time Reverse Transcription Polymerase Chain Reaction
SBM	Subjective Bayesian Model
Se	Sensitivity
Sp	Specificity
STROBE	Strengthening the Reporting of Observational Studies in Epidemiology
TN	True Negative
TP	True Positive
TRIPOD	Transparent Reporting of a Multivariable Prediction Model for Individual Prognosis or Diagnosis
WHO	World Health Organization
